# Synthesis of a Pseudodisaccharide α-*C*-Glycosidically Linked to an 8-Alkylated Guanine

**DOI:** 10.3390/molecules18043906

**Published:** 2013-04-02

**Authors:** Mu-Hua Huang, Jan Duchek, Andrea Vasella

**Affiliations:** 1 School of Materials Science and Engineering, Beijing Institute of Technology, Beijing 100081, China; 2 Laboratorium für Organische Chemie, Departement Chemie und Angewandte Biowissenschaften, ETH Zürich, HCI, Zürich CH-8093, Switzerland

**Keywords:** guanofosfocin, 8-alkylated guanine, ether-linked pseudodisaccharide, analogues, α-*C*-glycoside, nitrosopyrimidine

## Abstract

The synthesis of stable guanofosfocin analogues has attracted considerable attention in the past 15 years. Several guanofosfocin analogues mimicking the three constitutional elements of mannose, ribose, and guanine were designed and synthesized. Interest in ether-linked pseudodisaccharides and 8-alkylated guanines is increasing, due to their potential applications in life science. In this article, a novel guanofosfocin analogue **6**, an ether-linked pseudodisaccharide connected α-*C*-glycosidically to an 8-alkylated guanine, was synthesized in a 10-longest linear step sequence from known diol **13**, resulting in an overall yield of 26%. The key steps involve the ring-opening of cyclic sulfate **8** by alkoxide generated from **7** and a reductive cyclization of 4-*N*-acyl-2,4-diamino-5-nitrosopyrimidine **19** to form compound **6**.

## 1. Introduction

Stable carbohydrate mimics are used widely to study the biological functions of oligo- and polysaccharides [1,2,3]. Well-known examples include *C*-glycosides [4], carbasugars [5] and thiooligosaccharides [6].

**Figure 1:**
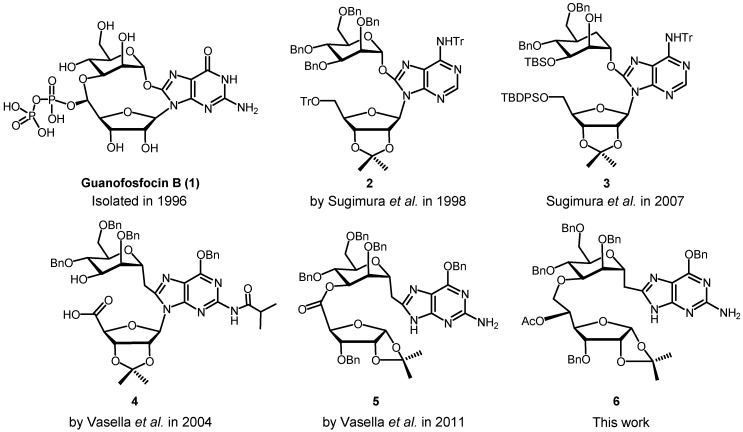
Compounds prepared on the way to stable analogues of guanofosfocins.

Guanofosfocin B (**1**, Figure 1) is one of the three guanofosfocins which were isolated in 1996 by Nippon Roche [7] from the fermentation broth of *Streptmyces* sp. AB 2570 and *Trichoderma* sp. FD 5372. Guanofosfocins are of interest as strong inhibitors of chitin synthases (IC_50_: 1–10 nM). Detailed investigations of their biological activity were, however, hampered by their rapid decomposition. The instability, not surprising considering the two activated acetal moieties found in **1**, was addressed by several groups by synthesizing stable open chain analogues of **1** while potentially retaining the promising biological properties. Sugimura *et al.* designed several analogues such as **2** (Figure 1) that maintained the alkoxy substituent attached to the C(8) of guanine [8,9,10]. Later on, they replaced the mannosyl moiety by a carba-mannosyl unit, and synthesized analogue **3** (Figure 1) [11]. Vasella *et al.* aimed at *C*-mannosides, replacing the anomeric oxygen by a methylene group, and prepared analogues **4** [12] and **5** [13] (Figure 1). We also considered the guanofosfocin analogue **6** of interest, in analogy to other, stable ether-linked pseudo-disaccharides [14,15,16] and report a synthesis of this ether-linked pseudo-disaccharide containing an 8-alkylated guanine.

## 2. Results and Discussion

**Scheme 1:**
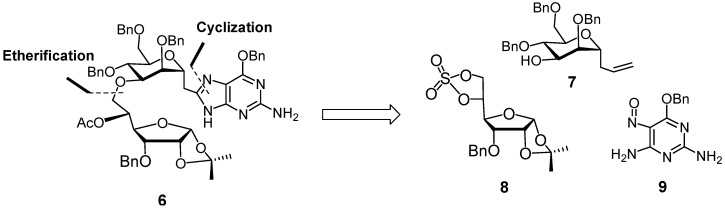
Retrosynthesis of pseudodisaccharide-guanine hybrid **6**.

The structure of **6** is characterized by an ether-linked pseudo-disaccharide with the α-*C*-mannopyranosyl unit linked to C(8) of a guanine via a methylene group. Accordingly, we had to incorporate a methylene group between the guanyl and mannosyl moieties and install the ether bond between the secondary C(3)-OH group of mannose and the C(6)-OH group of an allofuranose. Retrosynthetically (Scheme 1), the ether bond could be formed by ring-opening cyclic sulfate **8** by the alkoxy anion corresponding to alcohol **7** [17], and the 8-substituted guanine could be formed by regioselective 4*-N-*acylation of 2,4-diamino-5-nitrosopyrimidine **9**, followed by reductive cyclization, using a procedure developed by Vasella *et al.* [18]*.*

**Scheme 2:**
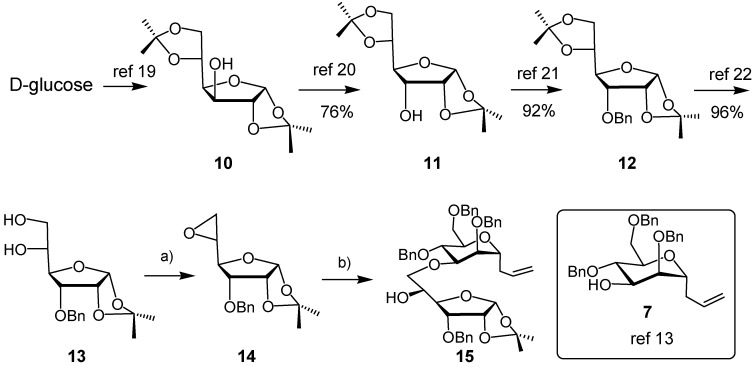
Synthesis of ether-linked pseudodisaccharide **15**.

Our synthesis started with the preparation of the protected α-allofuranose-diol **13**, following known procedures as outlined in Scheme 2 [19,20,21,22]. The secondary alcohol **7** was synthesized from commercially available methyl α-d-mannoside in 36% overall yield using the three-step sequence reported by Vasella *et al.* [13]. Epoxide formation from vicinal diol **13** under Mitsunobu conditions [23] yielded 66% of **14**. With epoxide **14** in hand, we carried out the oxirane ring-opening by the secondary alkoxide of **7** to get the pseudo-disaccharide **15** in 39% yield, besides 49% of recovered epoxide **14**.

*Reagents and conditions*: (a) Ph_3_P, DIAD, toluene, 66%; (b) **7**, NaH, DMF, 39%.

**Scheme 3:**
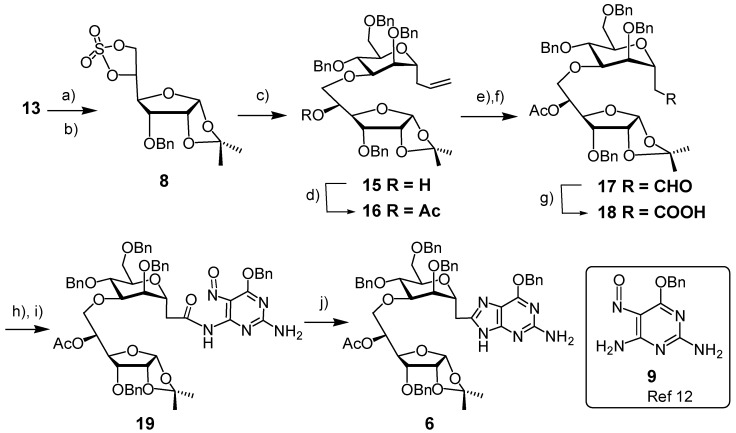
Synthesis of pseudodisaccharide-guanine hybrid **6** from cyclic sulfate **8**.

To improve the yield of the etherification, we prepared the cyclic sulfate **8** (Scheme 3). It was obtained in 69% from diol **13** by treatment with thionyl chloride and subsequent oxidation with NaIO_4_ in the presence of catalytic Ru(II)Cl_2_·xH_2_O [24]. The cyclic sulfate **8** was a colorless solid that darkened upon storage, even in the refrigerator (0–5 °C) and so was used immediately. The reaction between the cyclic sulfate **8** and the oxyanion derived from alcohol **7** in HMPA/THF took place smoothly to furnish the ether-linked pseudo-disaccharide **15** in a yield of 86% upon acidic aqueous work-up. The polar solvent proved crucial for the high yield. Alcohol **15** was acetylated and converted to the carboxylic acid **18** in a yield of 88% by dihydroxylation of **16** by OsO_4_/NMO, cleavage of the resulting diol by NaIO_4_, and oxidation of the resulting aldehyde by NaH_2_PO_4_/NaClO_2_. Treatment of acid **18** with oxalyl chloride in the presence of catalytic DMF furnished the acid chloride. Though the reaction was carried out for 1 h, the conversion was completed within 5 min, as observed by monitoring the reaction mixture by IR spectroscopy. The acid chloride was stable enough to allow routine characterization (IR, ^1^H-NMR and ^13^C-NMR). It reacted with 6-(benzyloxy)-5-nitrosopyrimidine-2,4-diamine (**9**) to afford amide **19**, accompanied by a color change from purple to blue-green. As purification of amide **19** by chromatography led to yields below 50%, presumably caused by a strong absorption of amide **19** on silica gel, the crude amide **19** was treated directly with triphenylphosphine in xylene under reflux to furnish guanine **6** in an overall yield of 50% from **18** (Scheme 3). The reductive cyclisation was accompanied by a color change from blue-green to brown.

Reagents and conditions: (a) SOCl_2_, Pyr., CH_2_Cl_2_; (b) RuCl_2_.xH_2_O, NaIO_4_, 69% for 2 steps; (c) **7**, NaH, HMPA/THF, 86%; (d) Ac_2_O, DMAP, Et_3_N; (e) OsO_4_, NMO; (f) NaIO_4_; (g) NaH_2_PO_4_, NaClO_2_, 88% for 4 steps; (h) (COCl)_2_, DMF(cat.), CH_2_Cl_2_; (i) **9**, Pyr., THF; (j) Ph_3_P, xylene, reflux; 50% for 3 steps.

With guanine **6** in hand, we performed a few scouting reactions to test its macrocyclization reactions via direct intra-molecular *N*-glycosylation. The first results showed that acetonide in **6** was not a good glycosyl donor for *N*-glycosylation. We then hydrolysed **6** to its corresponding vicinal diol. The macrocyclization is under investigation and the results will be reported in due course.

## 3. Experimental

General

Commercially available reagents were used without further purification. Water-free solvents were dried: THF was distilled from Na/benzophenone; toluene from Na; CH_2_Cl_2_, MeCN, MeOH, pyridine, and triethylamine from CaH_2_; acetone, and chloroform were dried over 4 Å molecular sieves. Technical solvents were distilled: AcOEt, CH_2_Cl_2_ from K_2_CO_3_; Et_2_O from FeSO_4_∙7 H_2_O; cyclohexane, hexane, MeOH, and toluene without any other additive. The reactions were carried out in oven-dried glassware, under an N_2_ or Ar atmosphere, unless stated otherwise. Qualitative TLC: precoated silica-gel plates (Merck silica gel 60 F_254_); detection by heating with ‘mostain’ (400 mL of 10% H_2_SO_4_ soln., 20 g of (NH_4_)_6_Mo_7_O_24_∙4H_2_O, 0.4 g of Ce(SO_4_)_2_∙4 H_2_O), or by UV. FCC (flash column chromatography): silica gel Fluka 60 (0.04–0.063 mm) or Merck silica gel *60* (0.063–0.200 mm) under slightly elevated pressure (0.1–0.4 bar). Melting points were measured on a Büchi B-540 melting point apparatus using open glass capillaries and are uncorrected. Optical rotations were measured with a PerkinElmer digital polarimeter: 1-dm cell at 25 °C, 589 nm, concentration (*c*) in g/100 mL. Infrared spectroscopy (IR) were recorded on a Perkin Elmer Spectrum RX-I FT-IR: *ca.* 2% soln. in CHCl_3_; absorptions in cm^–1^. NMR-spectra were recorded on Bruker magnetic resonance spectrometer (^1^H at 300 MHz, ^13^C at 75 MHz): chemical shifts δ in ppm relative to a residual undeuterated solvent peak. MS spectra were recorded on an IONSPEC Ultima ESI-FT-ICR spectrometer at 4.7 T.

*3-O-Benzyl-1,2-O-isopropylidene-α-D-allofuranose, 5,6-cyclic sulfate* (**8**). A solution of thionyl chloride (0.11 mL, 1.5 mmol) in CH_2_Cl_2_ (0.85 mL) was added dropwise to an ice-cooled solution of diol **13** (230 mg, 0.74 mmol) in CH_2_Cl_2_ (5 mL) and pyridine (0.24 mL, 3 mmol). The mixture was stirred for 5 min, when TLC revealed the disappearance of starting material. The mixture was diluted with CH_2_Cl_2_ and washed with water. The combined aqueous layers were extracted with CH_2_Cl_2_. The combined organic layers were dried (Na_2_SO_4_) and concentrated. The residue was dissolved in CH_2_Cl_2_/MeCN/H_2_O (2/2/3), to which was added NaIO_4_ (320 mg, 1.5 mmol) followed by Ru(II)Cl_2_∙xH_2_O (10 mg). After 10 min, the mixture was diluted with CH_2_Cl_2_, the organic layer was separated, the water layer was extracted with CH_2_Cl_2_. The combined organic layers were dried (Na_2_SO_4_) and concentrated *in vacuo*. The residue was purified by Flash Column Chromatography (FCC, EtOAc/cyclohexane, 1/3→1/1) to afford the cyclic sulfate **8** as a white solid (191 mg, 69%). m.p. 120–122 °C (dec.) (EtOAc/hexane). ^1^H-NMR (CDCl_3_, 300 MHz): δ (ppm) 7.40–7.37 (m, 5 H), 5.74 (d, 1 H, *J =* 3.3 Hz), 5.17 (dt, 1 H, *J =* 2.4, 7.2 Hz), 4.79 (dd, 1 H, *J =* 7.8, 9.0 Hz), 4.75 (d, 1 H, *J* = 12.0 Hz), 4.62 (d, 1 H, *J* = 12.0 Hz), 4.60 (d, 1 H, *J =* 6.9 Hz), 4.57 (dd, 1 H, *J =* 1.2, 5.4 Hz), 4.23 (dd, 1 H, *J =* 2.4, 9.0 Hz), 3.99 (dd, 1 H, *J =* 4.5, 9.0 Hz), 1.59 (s, 3 H), 1.36 (s, 3 H). ^13^C-NMR (CDCl_3_, 75 MHz): δ (ppm) 136.51, 128.53, 128.36, 113.53, 103.95, 79.99, 77.13, 77.05, 76.41, 72.59, 67.86, 26.90, 26.46. HR-MS(ESI), *m/z* 395.07695 [M+Na]^+^ (C_16_H_20_NaO_8_S^+^, required 395.07711).

*5,6-Anhydro-3-O-benzyl1,2-O-isopropylidene-α-D-allofuranose* (**14**). To a solution of diol **13** (305 mg, 0.98 mmol) and triphenylphosphine (310 mg, 1.18 mmol) in dry toluene (6 mL) was added diisopropyl azodicarboxylate (0.25 mL, 94% pure, 1.18 mmol) dropwise at room temperature. The mixture was stirred under reflux overnight. The solvent was removed *in vacuo*, the residue was purified by FCC (EtOAc/cyclohexane, 1/3) to give epoxide **14** as a colorless oil (189 mg, 66%). IR (Film, cm^−1^): 3021 (s), 2934 (w), 2870 (w), 1751 (w), 1455 (w), 1384 (w), 1375 (w), 1315 (w), 1254 (w), 1163 (w), 1131 (m), 1102 (s), 1022 (s). ^1^H-NMR (CDCl_3_, 300 MHz): δ (ppm) 7.40–7.29 (m, 5 H), 5.75 (d, 1 H, *J =* 3.9 Hz), 4.75 (d, 1 H, *J =* 11.4 Hz), 4.59–4.55 (m, 2 H), 4.21 (dd, 1 H, *J =* 3.0, 9.0 Hz), 3.66 (dd, 1 H, *J =* 4.2, 8.7 Hz), 3.19 (dd, 1 H, *J =* 3.0, 7.2 Hz), 2.81–2.73 (m, 2 H), 1.59 (s, 3 H), 1.36 (s, 3 H). ^13^C-NMR (CDCl_3_, 75 MHz): δ (ppm) 137.26, 128.57, 128.20, 128.16, 113.12, 104.21, 77.73, 77.57, 77.54, 72.02, 50.70, 44.45, 26.87, 26.60. [α]25 D +85.92° (*c* 1.05 in CHCl_3_).

*2,6-Anhydro-1,3,5-tri-*O*-benzyl-4-*O*-(3-*O*-benzyl-1,2-*O*-isopropylidene-6-deoxy-α-*d*-allofuranos-6-yl)-7,8,9-trideoxy-d-glycero-d-mannonon-8-enitol* (**15**). To a mixture of NaH (60% in oil, 93 mg, 2.32 mmol) in HMPA (3 mL) and THF (1 mL) was added alcohol **7** (1.10 g, 2.32 mmol) in THF (15 mL). The resulting slightly yellow solution was stirred at room temperature for 20 min, and then treated with cyclic sulfate **8** (720 mg, 1.93 mmol) in THF (5 mL). The resulting mixture was stirred at room temperature overnight when TLC revealed the disappearance of the cyclic sulfate. The reaction mixture was treated with H_2_SO_4_/THF/H_2_O (1/100/0.3, 3 mL), stirred for 1 h, treated with saturated aqueous NaHCO_3_ solution, and extracted with EtOAc. The combined organic layers were dried (MgSO_4_), concentrated, and the residue purified by FCC (EtOAc/cyclohexane, 1/2→1/1) to afford 15 as a colorless oil (1.28 g, 86%). IR (Film, cm^−1^): 3475 (w), 3067 (w), 3032 (w), 3009 (m), 2928 (m), 2872 (w), 1732 (w), 1672 (w), 1496 (w), 1454 (w), 1374 (w), 1313 (w), 1224 (m), 1094 (s), 1027 (s). ^1^H-NMR (CDCl_3_, 300 MHz): δ (ppm) 7.34–7.23 (m, 20 H), 5.84–5.70 (m, 1 H), 5.72 (d, 1 H, *J =* 3.3 Hz), 5.08–5.01 (m, 2 H), 5.72–4.48 (m, 9 H), 4.05–3.91 (m, 4 H), 3.86–3.48 (m, 8 H), 2.88 (d, 1 H, *J =* 2.4 Hz), 2.38–2.25 (m, 2 H), 1.57 (s, 3 H), 1.35 (s, 3 H). ^13^C-NMR (CDCl_3_, 75 MHz): δ (ppm) 138.22, 137.94, 137.90, 137.40, 134.16, 128.29, 128.19, 127.94, 127.87, 127.64, 127.39, 117.17, 112.86, 104.03, 78.66, 78.13, 77.71, 77.60, 75.14, 74.94, 73.86, 73.55, 73.29, 72.28, 72.13, 71.81, 71.10, 70.29, 68.96, 34.61, 26.93, 26.67. ([α]25 D +45.45° (*c* 1.00 in CHCl_3_). HR-MS (ESI), *m/z* 789.36095 [M+Na]^+^ (C_46_H_54_NaO_10_^+^, required 789.36092).

*2,6-Anhydro-1,3,5-tri-O-benzyl-4-O-(5-O-acetyl-3-O-benzyl-1,2-O-isopropylidene-6-deoxy-α-d-allofuranos-6-yl)-7,8,9-trideoxy-d-glycero-d-mannonon-8-enitol* (**16**). Triethylamine (0.32 mL, 2.29 mmol) and DMAP (61 mg, 0.50 mmol) in dry CH_2_Cl_2_ (2 mL), were added dropwise at 0 °C to a solution of alcohol **15** (0.88 g, 1.15 mmol) and acetic anhydride (0.21 mL, 2.29 mmol) in dry CH_2_Cl_2_ (3 mL). The resulting mixture was stirred at room temperature overnight. The reaction was quenched by adding water (1 mL), and the solvent was removed *in vacuo*. A solution of the residue in EtOAc (20 mL) was washed with water and 0.1 N HCl. The aqueous layer was extracted with EtOAc, the combined organic layers were dried (MgSO_4_) and concentrated to give the acetate **16** as a slightly yellow oil (0.94 g, 100%). IR (cm^−1^, CHCl_3_): 3067 (w), 3032 (w), 3010 (m), 2928 (w), 1738 (s), 1642 (w), 1496 (w), 1374 (m), 1374 (m), 1307 (w), 1240 (s), 1093 (s), 1027 (s). ^1^H-NMR (CDCl_3_, 300 MHz): δ (ppm) 7.34–7.26 (m, 20 H), 5.85–5.73 (m, 1 H), 5.64 (d, 1 H, *J =* 3.7 Hz), 5.31 (ddd, 1 H, *J =* 4.8, 3.0, 6.6 Hz), 5.11–5.04 (m, 2 H), 4.73 (d, 1 H, *J =* 11.3 Hz), 4.67 (d, 1 H, *J =* 11.2 Hz), 4.61–4.40 (m, 7 H), 4.12 (dd, 1 H, *J =* 5.3, 8.6 Hz), 4.05–3.98 (m, 1 H), 3.90 (dd, 1 H, *J =* 4.3, 8.9 Hz), 3.83–3.62 (m, 8 H), 2.41–2.05 (m, 2 H), 1.85 (s, 3 H), 1.53 (s, 3 H), 1.31 (s, 3 H). ^13^C-NMR (CDCl_3_, 75 MHz): δ (ppm) 170.27 (CO), 138.57, 138.45, 138.37, 137.50, 128.40, 128.38, 128.36, 128.29, 128.14, 127.99, 127.83, 127.74, 127.63, 127.58, 127.43, 117.27, 113.08, 104.17, 79.81, 78.99, 77.20, 76.46, 75.53, 75.22, 73.93, 73.57, 73.97, 73.57, 73.28, 72.84, 72.21, 71.92, 71.75, 69.28, 68.92, 34.43, 26.80, 26.60, 21.00. [α]25 D +43.73° (*c* 1.65 in CHCl_3_). HR-MS (ESI), *m/z*, 831.37633 [M+Na]^+^ (C_48_H_56_NaO_11_^+^, requires 831.37148).

*2-[3-O-(5-O-Acetyl-3-O-benzyl-1,2-O-isopropylidene-6-deoxy-α-d-allofuranos-6-yl)-2,4,6-tri-O-benzyl-α-D-mannopyranosyl]acetaldehyde* (**17**). To a mixture of alkene **16** (0.850 g, 1.05 mmol) and *N*-methylmorpholine *N*-oxide (NMO) (213 mg, 1.58 mmol) in acetone (6 mL) and water (2 mL) was added osmium tetroxide (0.2 w% in water, 2.6 mL, 0.021 mmol, 0.02 eq.) at 0 °C. The resulting mixture was stirred for 45 h when TLC revealed the disappearance of the alkene **16**. Upon addition of sodium sulfite (1.12 g) the yellow suspension turned into a slightly yellow two-layered solution. The upper organic layer was separated, the aqueous layer was extracted with EtOAc, the combined organic layers were dried (MgSO_4_) and concentrated to give the crude diol (802 mg, 91%) as a mixture of two epimers in a ratio of ca. 0.75:1 (based on the integrals in the ^1^H-NMR spectrum). IR (cm^−1^, CHCl_3_): 3473 (w), 3031 (vw), 2931 (w), 2871 (w), 1737 (m), 1496 (w), 1454 (m), 1372 (m), 1236 (s), 1164 (w), 1090 (s), 1070 (s), 1024 (s). ^1^H-NMR (CDCl_3_, 300 MHz): δ (ppm) 7.32–7.24 (m, 32.3 H), 5.63 (d, 0.6 H, *J =* 3.0 Hz), 5.60 (d, 0.8 H, *J =* 3.3 Hz), 5.29–5.23 (m, 1.5 H), 5.67–4.39 (m, 13.9 H), 4.17–4.06 (m, 4.0 H), 3.95–3.78 (m, 7.2 H), 3.72–3.42 (m, 13.9 H), 4.17–4.06 (m, 4.0 H), 3.95–3.78 (m, 7.2 H), 3.72–3.42 (m, 13.9 H), 2.04 (d, 1.2 H, *J =* 0.9 Hz), 1.84–1.83 (m, 5.1 H), 1.78–1.51 (m, 2.9 H), 1.52 (s, 5.3 H), 1.31 (br s, 5.2 H). ^13^C-NMR (CDCl_3_, 75 MHz): δ (ppm) 170.29, 138.17, 138.10, 138.07, 138.00, 137.98, 137.49, 137.46, 128.46, 128.42, 128.16, 128.14, 128.02, 127.94, 127.90, 127.83, 127.80, 127.74, 127.70, 127.66, 133.11, 133.09, 104.15, 79.84, 77.20, 76.79, 76.67, 76.40, 76.31, 75.25, 73.61, 73.45, 73.42, 73.18, 71.93, 71.80, 69.47, 69.39, 66.38, 32.81, 32.69, 26.82, 26.60, 21.01. HR-MS(ESI), *m/z*, 865.37635 [M+Na]^+^ (C_48_H_58_NaO_13_^+^, required 865.37696).

To the diol (630 mg, 0.747 mmol) in MeOH (3 mL) and H_2_O (5 mL) was added sodium periodate (190 mg, 0.897 mmol) in H_2_O (3 mL) at 0 °C, the mixture was stirred for 60 min, and then extracted with EtOAc. The combined organic layers were dried (Mg_2_SO_4_) and concentrated to give the aldehyde **17** as a slightly yellow oil which was pure according to the NMR spectrum. IR (cm^−1^, CHCl_3_): 3030 (vw), 2933 (w), 2870 (w), 1739 (m), 1726 (m), 1496 (w), 1454 (w), 1371 (m), 1307 (m), 1234 (s), 1091 (s), 1071 (s), 1025 (s). ^1^H-NMR (CDCl_3_, 300 MHz): δ (ppm) 9.68 (t, 1 H, *J =* 2.3 Hz), 7.36–7.21 (m, 20 H), 5.63 (d, 1 H, *J =* 3.7 Hz), 5.28 (ddd, 1 H, *J =* 4.6, 3.4, 6.4 Hz), 4.74–4.42 (m, 10 H), 4.13 (dd, 1 H, *J =* 5.2, 8.9 Hz), 3.96–3.61 (m, 9 H), 2.62 (dd, 1 H, *J =* 1.1, 2.0 Hz), 2.60 (t, 1 H, *J =* 2.3 Hz), 1.87 (s, 3 H), 1.55 (s, 3 H), 1.34 (s, 3 H). ^13^C-NMR (CDCl_3_, 75 MHz): δ (ppm) 200.50, 170.21, 138.40, 138.07, 137.92, 137.79, 137.54, 129.09, 128.50, 128.48, 128.44, 128.38, 128.11, 128.08, 128.03, 127.90, 127.87, 127.82, 127.79, 127.57, 125.36, 113.11, 104.17, 79.80, 77.21, 76.52, 76.43, 76.00, 74.91, 74.43, 73.24, 72.85, 72.18, 71.80, 71.49, 69.65, 68.36, 66.57, 45.32, 26.53, 26.62, 21.52, 21.00. [α]25 D +56.27° (*c* 2.00 in CHCl_3_). HR-MS(ESI), *m/z*, 833.35133 [M+Na]^+^ (C_4__7_H_5__4_NaO_1__2_^+^, required 833.35075).

*2-[3-O-(5-O-Acetyl-3-O-benzyl-1,2-O-isopropylidene-6-deoxy-α-d-allofuranos-6-yl)-2,4,6-tri-O-benzyl-α-D-mannopyranosyl]acetic acid* (**18**). The crude aldehyde **17** in acetonitrile (6 mL) was treated with NaH_2_PO_4_ (34 mg, 0.25 mmol) in H_2_O (2 mL) and H_2_O_2_ (30%, 0.12 mL, 1.12 mmol), respectively, at 0 °C, followed by addition NaClO_2_ (0.17 g, 80%, 1.49 mmol) in H_2_O (2 mL) at 0 °C. The resulting mixture was stirred overnight, brought to pH 2.0 with 1N HCl, and extracted with EtOAc. The combined organic layers were dried (MgSO_4_) and concentrated to give acid **18** as a colorless gum (0.60 g, 88% from **16**). IR(cm^−1^, CHCl_3_): 3062 (w), 3030 (w), 2933 (w), 2872 (w), 1739 (s), 1714 (m), 1496 (w), 1454 (w), 1372 (m), 1306 (w), 1236 (s), 1162 (m), 1095 (s), 1026 (s). ^1^H-NMR (CDCl_3_, 300 MHz): δ (ppm) 7.33–7.25 (m, 20 H), 5.61 (d, 1 H, *J =* 3.6 Hz), 5.23 (dd, 1 H, *J =* 5.3, 10.2 Hz), 4.68–4.36 (m, 10 H), 4.34–4.30 (m, 1 H), 4.10 (dd, 1 H, *J =* 5.3, 8.9 Hz), 3.97 (br. s, 1 H), 3.89–3.78 (m, 3 H), 3.73 (d, 1 H , *J =* 2.2 Hz), 3.64–3.56 (m, 3 H), 2.70 (dd, 1 H, *J =* 4.3, 15.7 Hz), 2.56 (dd, 1 H, *J =* 8.6, 15.9 Hz), 1.84 (s, 3 H), 1.52 (s, 3 H), 1.31 (s, 3 H). ^13^C-NMR (CDCl_3_, 75 MHz): δ (ppm) 175.43, 170.35, 138.31, 138.06, 137.88, 137.50, 128.47, 128.43, 128.36, 128.17, 128.04, 127.97, 127.89, 127.86, 127.81, 127.56, 133.13, 104.17, 79.80, 77.20, 76.86, 76.45, 75.66, 74.84, 74.47, 73.33, 73.05, 72.24, 71.84, 71.50, 69.45, 68.40, 68.16, 36.25, 26.98, 26.81, 26.61, 20.98. [α]25 D +50.97° (*c* 4.52 in CHCl_3_). HR-MS(ESI), *m/z*, 849.34625 [M+Na]^+^ (C_47_H_54_NaO_13_^+^, requires 849.34621), 871.32809 [M−H+Na_2_]^+^ (C_47_H_534_Na_2_O_13_^+^, requires 871.32816).

*2-[3-O-(5-O-Acetyl-3-O-benzyl-1,2-O-isopropylidene-6-deoxy-α-d-allofuranos-6-yl)-2,4,6-tri-O-benzyl-α-D-mannopyranosyl]-N-[2-amino-6-(benzyloxy)-5-nitrosopyrimidin-4-yl]acetamide* (**19**). To a solution of acid **18** (0.470 g, 0.57 mmol) in CH_2_Cl_2_ (4 mL) was added oxalyl chloride (0.15 mL, 1.7 mmol) followed by a drop of DMF at 0 °C. The resulting mixture was stirred for 1 h and concentrated in vacuo. The crude acid chloride was pure according to its NMR spectra and used directly for next step. IR (cm^−1^, CHCl_3_): 3031 (vw), 2932 (vw), 2868 (vw), 1798 (m), 1740 (s), 1496 (w), 1454 (m), 1371 (m), 1234 (s), 1091 (s), 1069 (s), 1023 (s). ^1^H-NMR (CDCl_3_, 300 MHz): δ (ppm) 7.36–7.24 (m, 20 H,), 5.61 (d, 1 H, *J* = 3.7 Hz), 5.23 (dd, 1 H, *J* = 6.0, 10.0 Hz), 4.95 (d, 1 H, *J =* 11.0 Hz), 4.73–4.31 (m, 9 H), 4.10 (dd, 1 H, *J* = 5.4, 8.9 Hz), 3.97 (t, 1 H, *J* = 5.8 Hz), 3.86 (dd, 1 H, *J* = 4.3, 8.8 Hz), 3.81–3.67 (m, 4 H), 3.64–3.57 (m, 3 H), 3.21–3.14 (m, 1 H), 2.99–2.90 (m, 1 H), 1.84 (s, 3 H), 1.52 (s, 3 H), 1.31 (s, 3 H). ^13^C-NMR (CDCl_3_, 75 MHz): δ (ppm) 171.27, 170.22, 138.39, 137.93, 137.54, 137.48, 128.55, 128.48, 128.34, 128.16, 128.02, 127.99, 127.84, 127.77, 127.52, 113.12, 104.13, 79.89, 77.32, 77.23, 76.30, 75.93, 75.25, 74.78, 74.64, 73.34, 72.56, 72.23, 71.74, 71.21, 69.72, 68.25, 67.23, 49.09, 26.82, 26.61, 20.95, 13.97. [α]25 D +43.68° (*c* 2.35 in CHCl_3_).

To a solution of 2,4-diamino-5-nitrosopyrimidine **9** (0.21 g, 0.86 mmol) and pyridine (0.07 mL) in dry THF (12 mL) was added the above acid chloride (0.47 g, 0.57 mmol) in THF (7 mL) at 0 °C dropwise. Once the acid chloride was added, the deep-blue solution turned green. After stirring for 1 h, the purple solid was filtered off, and the filtrate was concentrated *in vacuo* to afford the amide **19** (600 mg). IR (cm^−1^, CHCl_3_): 3328 (w), 3228 (w), 3031 (w), 2933 (w), 2870 (w), 1739 (m), 1631 (m), 1598 (s), 1535 (s), 1497 (m), 1454 (s), 1371 (m), 1347 (s), 1255 (s), 1209 (s), 1091 (s), 1072 (s), 1026 (s). ^1^H-NMR (CDCl_3_, 300 MHz): δ (ppm) 12.38 (br. s, 1 H, exchange with D_2_O), 7.51–7.23 (m, 20 H), 7.17 (br. s, 1 H, exchange with D_2_O), 6.03 (br. s, 1 H, exchange with D_2_O), 5.65 (d, 1 H, *J =* 3.7 Hz), 5.63 (s, 2 H), 5.27 (dd, 1 H, *J =* 5.8, 10.0 Hz), 4.70 (d, 1 H, *J =* 11.0 Hz), 4.64 (d, 1 H, *J =* 11.6 Hz), 4.63 (d, 1 H, *J =* 11.8 Hz), 4.57–4.43 (m, 6 H), 4.14 (dd, 1 H, *J =* 5.5, 8.9 Hz), 4.06 (br. s, 1 H), 3.88 (dd, 1 H, *J =* 4.3, 8.9 Hz), 3.83–3.65 (m, 7 H), 2.97 (dd, 1 H, *J =* 5.3, 14.7 Hz), 2.89 (dd, 1 H, *J =* 7.6, 14.9 Hz), 1.83 (s, 3 H), 1.51 (s, 3 H), 1.32 (s, 3 H). ^13^C-NMR (CDCl_3_, 75 MHz): δ (ppm) 171.45, 170.35, 163.78, 138.91, 138.31, 138.04, 137.86, 137.46, 135.49, 128.64, 128.46, 128.44, 128.32, 128.26, 128.20, 128.06, 127.95, 127.80, 127.72, 127.51, 113.12, 104.15, 79.92, 77.33, 77.24, 76.20, 76.05, 74.74, 74.12, 73.13, 72.25, 71.91, 71.62, 69.40, 68.72, 68.62, 41.33, 26.72, 26.61, 20.97. [α]25 D +81.92° (*c* 0.053 in CHCl_3_). HR-MS (ESI), *m/z*, 1076.4270 [M+Na]^+^ (C_58_H_63_N_5_NaO_14_^+^, required 1076.4264).

*8-[(3-O-((5-O-Acetyl-3-O-benzyl-1,2-O-isopropylidene-6-deoxy-α-d-allofuranos-6-yl)-2,4,6-tri-O-benzyl-α-D-mannopyranosyl)methyl]-O^6^-benzylguanine* (**6**). A mixture of the amide **19** (600 mg, 0.57 mmol) and triphenyl phosphine (449 mg, 1.71 mmol) in xylene was stirred at 140 °C overnight and then cooled to room temperature. The mixture was passed through silica gel (EtOAc/cyclohexane 1/1→1/0) to give guanine 6 as a yellowish oil (291 mg, 50% from acid **18**). IR (cm^−1^, CHCl_3_): 3528 (w), 3420 (w), 3067 (w), 3010 (m), 2928 (m), 1738 (m), 1625 (s), 1591 (s), 1496 (w), 1455 (m), 1374 (m), 1325 (m), 1227 (s), 1146 (m), 1090 (s). ^1^H-NMR (CDCl_3_, 300 MHz): δ (ppm) 11.65 (br s, 1 H, NH, exchange with D_2_O), 7.50–7.20 (m, 25 H), 5.57 (d, 1 H, *J =* 3.7 Hz), 5.53 (s, 2 H), 5.18 (dd, 1 H, *J =* 5.0, 10.7 Hz), 4.80 (br. s, 2 H, exchange with D_2_O), 4.71–4.35 (m, 10 H), 4.21 (t, 1 H, *J =* 8.1 Hz), 4.08 (dd, 2 H, *J =* 5.0, 8.9 Hz), 3.86–3.70 (m, 3 H), 3.61 (dd, 1 H, *J =* 2.6, 7.7 Hz), 3.58–3.53 (m, 2 H), 3.47 (dd, 1 H, *J =* 4.0, 10.2 Hz), 3.30 (d, 1 H, *J =* 14.0 Hz), 2.97 (dd, 1 H, *J =* 10.8, 16.0 Hz), 1.84 (s, 3 H), 1.49 (s, 3 H), 1.30 (s, 3 H). ^13^C-NMR (CDCl_3_, 75 MHz): δ (ppm) 170.14, 160.10, 158.72, 155.66, 148.61, 137.74, 137.65, 137.42, 136.79, 132.20, 132.07, 128.61, 128.56, 128.49, 128.46, 128.40, 128.35, 128.32, 128.22, 128.04, 127.98, 127.92, 127.82, 127.69, 114.66, 13.06, 104.16, 79.68, 77.26, 77.11, 76.47, 75.95, 75.27, 74.37 (CH_2_), 73.14, 72.70, 72.15, 71.79, 69.86, 67.79, 26.95, 26.78, 26.55, 20.98. [α]25 D +31.76° (*c* 1.41 in CHCl_3_). HR-MS (ESI), *m/z*, 1044.4378 [M+Na]^+^ (C_58_H_63_N_5_NaO_12_^+^, required 1044.4365).

## 4. Conclusions

In summary, a new linear analogue of guanofosfocin **6** was synthesized in 10 steps from the known sugar diol **13** in 26% overall yield. Key steps were the ring-opening of cyclic sulfate **8** by a sugar *sec*-alkoxide and the reductive cyclization of 4-*N*-acyl-2,4-diamino-5-nitrosopyrimidine **19**. The robustness of the above etherification and of the reductive cyclization will find more applications in synthetic organic chemistry. The structure of **6** was characterized as an ether-linked pseudodisaccharide α-*C*-glycosidically linked to an 8-alkylated guanine. This compound, together with previously synthesized analogues of guanofosfocins, allows expansion of the relevant research in this field of medicinal chemistry and chemical biology.
